# Severe burn injury in europe: a systematic review of the incidence, etiology, morbidity, and mortality

**DOI:** 10.1186/cc9300

**Published:** 2010-10-19

**Authors:** Nele Brusselaers, Stan Monstrey, Dirk Vogelaers, Eric Hoste, Stijn Blot

**Affiliations:** 1 Department of General Internal Medicine, Infectious Diseases and Psychosomatic Medicine, Ghent University Hospital, De Pintelaan 185, Ghent 9000, Belgium; 2 Department of Plastic Surgery and Burn Unit, Ghent University Hospital, De Pintelaan 185, Ghent 9000, Belgium; 3 Faculty of Medicine and Health Sciences, Ghent University, De Pintelaan 185, Ghent 9000, Belgium; 4 Intensive Care Unit, Ghent University Hospital, De Pintelaan 185, Ghent 9000, Belgium; 5 Department of Healthcare, University College Ghent, Keramiekstraat 80, Ghent 9000, Belgium

## Abstract

**Introduction:**

Burn injury is a serious pathology, potentially leading to severe morbidity and significant mortality, but it also has a considerable health-economic impact. The aim of this study was to describe the European hospitalized population with severe burn injury, including the incidence, etiology, risk factors, mortality, and causes of death.

**Methods:**

The systematic literature search (1985 to 2009) involved PubMed, the Web of Science, and the search engine Google. The reference lists and the Science Citation Index were used for hand searching (snowballing). Only studies dealing with epidemiologic issues (for example, incidence and outcome) as their major topic, on hospitalized populations with severe burn injury (in secondary and tertiary care) in Europe were included. Language restrictions were set on English, French, and Dutch.

**Results:**

The search led to 76 eligible studies, including more than 186,500 patients in total. The annual incidence of severe burns was 0.2 to 2.9/10,000 inhabitants with a decreasing trend in time. Almost 50% of patients were younger than 16 years, and ~60% were male patients. Flames, scalds, and contact burns were the most prevalent causes in the total population, but in children, scalds clearly dominated. Mortality was usually between 1.4% and 18% and is decreasing in time. Major risk factors for death were older age and a higher total percentage of burned surface area, as well as chronic diseases. (Multi) organ failure and sepsis were the most frequently reported causes of death. The main causes of early death (<48 hours) were burn shock and inhalation injury.

**Conclusions:**

Despite the lack of a large-scale European registration of burn injury, more epidemiologic information is available about the hospitalized population with severe burn injury than is generally presumed. National and international registration systems nevertheless remain necessary to allow better targeting of prevention campaigns and further improvement of cost-effectiveness in total burn care.

## Introduction

Burn injury is a common type of traumatic injury, causing considerable morbidity and mortality. Moreover, burns are also among the most expensive traumatic injuries, because of long hospitalization and rehabilitation, and costly wound and scar treatment [1,2].

Worldwide, an estimated 6 million patients seek medical help for burns annually, but the majority are treated in outpatient clinics [3]. Whether inpatient treatment in a specialized burn unit is required depends principally on the severity of the burn, the concomitant trauma, and the general condition of the patient [4-7]. In the European Union, transport accidents (21.8%), accidental falls (19.4%), and suicide (24.7%) are the three most common "fatal injuries," with burns reported as "other unintentional fatal injuries," together with poisoning and drowning (34.1%) [8]. Exact European figures about severe burn injury are still unavailable, and most European countries do not yet have a national registration system of hospitalized patients with severe burn injury [9]. In the United States, burns due to fire and flames (fatal in 6.1%) and hot objects or substances (fatal in 0.6%) represent 2.4% of all trauma cases in the United States (based on hospital admissions and death registers) and are responsible for 1.6% of the traumatic deaths [10]. Published data vary considerably depending on the source(s) and classification system (ICD codes, W.H.O definitions, and so on) used and can therefore be extremely difficult to compare. The aim of this study was therefore to summarize the available European epidemiologic data, based on scientific studies in international journals, instead of (often inaccurate) nationwide estimates.

## Materials and methods

**Figure 1:**
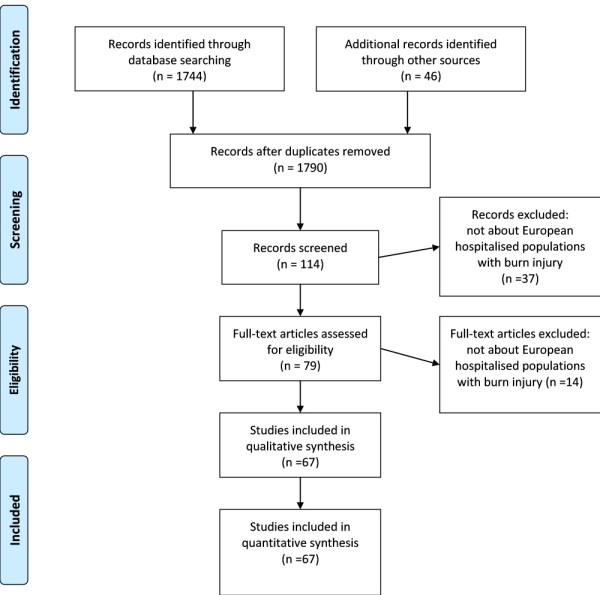
**PRISMA Flow Diagram: description of the literature search**.

This systematic literature search aimed to include all studies from 1985 until December 2009 reporting on etiology, incidence, prevalence, and/or outcome of severe burn injuries as the major topic [11], from all European states and territories, an area of more than 800 million inhabitants and ~250 specialized burn units (Table 1, Figure 1). "Severe" burn injury has been defined as an acute burn injury in need of specialized care during hospital admission. Because the definition of burn unit may be different nationally and internationally (for example, only high care, ...), and several countries did not have specialized burn units (at the start of our study period), we included all hospitalized burn populations. Therefore, the included populations could also be admitted to surgery and pediatric wards, general intensive care units, and so on. The first selection of the search was performed by one investigator (NB) under supervision of the principal investigators (SB, EH), who are content experts. Language restrictions were set to English, French, and Dutch. Studies only considering deceased patients with burn injury were excluded. Assessment of eligibility of the remaining articles (after exclusion of the irrelevant articles) was performed after mutual consideration. The PubMed search included automatic and manual search strategies with the following MeSH terms: 'burns,' 'epidemiology,' 'incidence,' 'fatal outcome,' 'mortality,' and 'causality,' which resulted in 1,744 hits, in the selected languages and within the selected study period (about humans). Therefore, more-specific combinations were used (for example, searching by country), also consulting the Web of Science, Google, and hand-searching reference lists and citation reports of the relevant articles.

**Table 1:** States and territories of Europe (as reported by the Population Reference Bureau, used by the United Nations when categorizing geographic subregions)

Country	**Population**^**a **^**(million)**	**HDI**^**d**^	Capital city or largest city
**Eastern Europe**			
Belarus	9.7	0.826	Minsk
^b^Bulgaria	7.6	0.840	Sofia
^b^Czech Republic	10.5	0.903	Prague
^b^Hungary	10.0	0.879	Budapest
Moldova	4.1	0.720	Chisinau
^b^Poland	38.1	0.880	Warsaw
^b^Romania	21.5	0.837	Bucharest
Russian Federation	141.8	0.817	Moscow
^b^Slovakia	5.4	0.880	Bratislava
Ukraine	46.0	0.796	Kiev

**Northern Europe**			
^b^Denmark	5.5	0.955	Copenhagen
^b^Estonia	1.3	0.883	Tallinn
^b^Finland	5.3	0.959	Helsinki
^c^Iceland	0.3	0.969	Reykjavik
^b^Ireland	4.5	0.965	Dublin (City)
^b^Latvia	2.3	0.866	Riga
^b^Lithuania	3.3	0.870	Vilnius
^c^Norway	4.8	0.971	Oslo
^b^Sweden	9.3	0.963	Stockholm
^b^United Kingdom	61.8	0.947	London

**Southern Europe**			
Albania	3.2	0.818	Tirana
Andorra	0.1	0.934	Andorra la Vella
Bosnia and Herzegovina	3.8	0.812	Sarajevo
Croatia (Hrvatska)	4.4	0.871	Zagreb
^b^Cyprus	1.1	0.914	Nicosia (Lefkosia)
^b^Greece	11.3	0.942	Athens
Vatican City State	0.001	-	Vatican City
^b^Italy	60.3	0.951	Rome, Milan (Metro)
Macedonia, Rep. of	2.0	0.817	Skopje
^b^Malta	0.4	0.902	Valletta
Montenegro	0.6	0.834	Podgorica
^b^Portugal	10.6	0.909	Lisbon
San Marino	0.03	-	San Marino
Serbia	7.3	0.826	Belgrade
^b^Slovenia	2.0	0.929	Ljubljana
^b^Spain	46.9	0.955	Madrid
Turkey	74.8	0.806	Ankara, Istanbul

**Western Europe**			
^b^Austria	8.4	0.955	Vienna (Wien)
^b^Belgium	10.8	0.953	Brussels
^b^France	62.6	0.961	Paris
^b^Germany	82.0	0.947	Berlin
^c^Liechtenstein	0.04	0.951	Vaduz
^b^Luxembourg	0.5	0.960	Luxembourg
Monaco	0.04	-	Monaco
^b^Netherlands	16.5	0.964	Amsterdam
^c^Switzerland	7.8	0.960	Bern, Zürich

^**a**^Population numbers mid 2009; ^b^member states of the European Union (EU); ^c^member states of European Free Trade Association (EFTA); ^d^HDI, Human Development Index (2009) [12]: three European microstates are not ranked in the 2009 HDI, for being unable or unwilling to provide the necessary data at the time of publication of the HDI ranking (although it could be expected to fall within the 'very high' HDI category).

### Data analysis

The following data were collected: (a) basic study characteristics: author, year of publication, study period, country, retrospectively or prospectively gathered data, number of participating centers; setting (burn unit, surgical department); (b) population characteristics: number of hospitalized patients with burn injury, analyzed subgroups (for example, military personal, immigrants), age group (all ages, only adult, pediatric or elderly population), exclusion criteria; (c) occurrence rate and outcome (including proportion hospitalized); (d) patient characteristics: mean/median age and total burned surface area (TBSA), inhalation, gender; (e) etiology: the etiology of the burns was reported in the following five groups: flames/explosion (also including fireworks, and so on), scalds/steam (also including burns caused by warm food and oil), contact burns, chemical burns, and electrical burns.

Because of the various ways of reporting in the different studies, the most common (and numeric) way of reporting was registered in our database. For certain variables, the most prevalent way of reporting was used for the analyses (for example, mean TBSA instead of median TBSA). For example, if TBSA was only reported graphically (by age group), this could not be used in our analysis. If variables were only reported separately for survivors and nonsurvivors, these variables were not used in the analyses, although they are reported in the main table (cf. Additional file 1). In case of different subgroups, the most 'normal' subgroup was used for the analyses (for example, if a subgroup of immigrants/military personnel was compared with 'native' civilians, only the latter were used in the analysis).

Because mean age and TBSA were provided in several studies, the correlation with mortality could be calculated with a one-tailed Pearson test, and correlation plots were made. A positive correlation reflected in a dependent variable (mortality) will increase if the independent variable (age, TBSA) increases. Box-plots were used to analyze and visualize the proportion of the different etiologies. Statistical analyses were performed with the software program SPSS for Windows, version 16 (SPSS Inc., Somers, NY).

A better standard of life and economy is expected to be related to better health care, which might consequently be related to differences in incidence, etiology, and outcome. Therefore, the studies were also grouped and classified by their Human Development Index (HDI) ranking of the countries [12]. The HDI measures development by combining indicators of life expectancy, educational attainment, and income into a composite HDI. The HDI is in fact a single statistic that serves as a frame of reference for both social and economic development [12]. The HDI sets a minimum and a maximum for each dimension, called goalposts, and then shows where each country stands in relation to these goalposts, expressed as a value between 0 and 1. All countries worldwide are categorized in four groups by their HDI: 'low' (<0.500), 'medium' (0.500-0.799), 'high' (0.800-0.899), and 'very high (0.900 and 1.000) [12].

## Results

We found 76 studies from 22 countries, of which 73 studies were published in English, and three studies, in French [13-15]. For the other European countries, no eligible studies were found. These studies include more than 186.500 patients in total (the total number of patients was not always reported) [1,13,14,16-58]. Of these studies, 20 studies considered only children (16 years or younger) [59-78], and 11, only patients of 60 to 75 years or older (described as 'elderly') [15,79-88] populations with severe burn injury (Table 2). The other 45 studies were analyzed together (and described as 'overall'). Additional file 1 gives an overview of the most important epidemiologic data available for each study.

**Table 2:** Number of included studies for each country

Region	Country	Number of studies	HDI (rank)
Eastern Europe	Czech Republic	6	.903 (36)^b^
	Hungary	1	.879 (43)^a^
	Romania	1	.837 (63)^a^
	Slovakia	2	.880 (42)^a^
Northern Europe	Denmark	4	.955 (16)^b^
	Finland	4	.959 (12)^b^
	Iceland	1	.969 (3)^b^
	Ireland	2	.965 (5)^b^
	Lithuania	1	.870 (46)^a^
	Norway	2	.971 (1)^b^
	United Kingdom	14	.947 (21)^b^
	Sweden	1	.963 (7)^b^
Southern Europe	Greece	1	.942 (25)^b^
	Italy	2	.951 (18)^b^
	Portugal	1	.909 (34)^b^
	Spain	12	.955 (15)^b^
	Turkey	3	.806 (79)^a^
Western Europe	Austria	3	.955 (14)^b^
	Belgium	2	.953(17)^b^
	France	7	.961 (8)^b^
	Germany	2	.947 (22)^b^
	The Netherlands	4	.964 (6)^b^

**Total**		76	

The Human Development Index (HDI) Ranking is a classification of all countries worldwide based on life expectancy, literacy, education, and standards of living. Higher numbers are related to a higher development index (*^a ^'high' HDI, **^b ^'very high' HDI).

### Occurrence rate

Of all patients presenting in the emergency department with burns, between 4% and 22% were hospitalized in (intensive care) burn units [2,19-21,24,27,28]. The annual incidence of patients with severe burn injury was reported in 22 studies and lies between 0.2 and 2.9/10,000 inhabitants. In one Lithuanian study, the incidence was remarkably higher (6.6 in 1991, which decreased to 4.0 in 2004) [57]. It was higher among children, even up to 8.3/10,000 for children younger than 5 years in one Norwegian study [56], and 8.5/10,000 for all children younger than 15 years in a Czech study [75]. A higher incidence has been associated with a lower standard of life and ethnic minorities [61,62,65].

The incidence has decreased over the last 30 years. This was usually reported as the annual number of admitted patients (without denominator), or only graphically. This decreasing trend is (almost) linear, but the decline became less steep since the early 1990s [24,40,50]. In Slovakia, a 20% reduction of the number of patients was reported between 1990 and 2004 [22]. The decrease was reported to be present in all age groups [85], but in one Danish study (1987), it was almost exclusively due to a reduction of burns in children younger than 5 years [16]. Another Danish study (1986) reported that the decline is mainly due to a decrease in number of accidents at work [26]. Only two (Icelandic and Czech) studies reported an increasing incidence of pediatric burns [59,75], which was, in Iceland, associated with the increased domestic use of geothermal water (≥70°C) [59].

### Age and gender distribution

Children account for almost half of the population with severe burn injury (40% to 50%) [14,16,25,34,41,44,58,63,65]. In one study from Turkey, only 25% were adults [30]. Children younger than 5 years account for 50% to 80% of all childhood burns [14,32,41,50,64,72,74,78]. The growth of the elderly population in the Western world is also reflected in the hospitalized population with severe burn injury, by an increasing mean age, or by an increased proportion of elderly (10% to 16% of the total population with severe burn injury) [14,33,41,50,58,79-81,83-87,89].

In most studies, an overall male predominance of 55% to 75% was described. This may be explained by the fact that burn injuries in adults are often work related [2]. In one Austrian and one Turkish study, only one third were men, but this dissimilarity was not discussed or explained in these articles. In the pediatric populations, 60% to 65% are boys, but in the elderly population, a female predominance of up to 65% was found, which might be related to the higher life expectancy in the female gender.

### Etiology and circumstances of the accident

**Figure 2:**
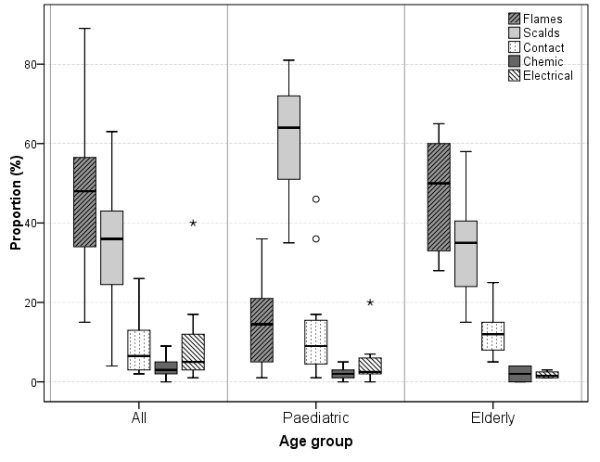
**Etiology of severe burn injury, according to the age group (proportion of all burns)**. Forty-one studies provided sufficient data to compare the etiologies. In the 'All' group, two of the 19 studies consider only adults. The 'pediatric' box plots are based on 14 studies; the 'elderly' box plots, on eight studies.

Flames, scalds (including steam), and contact burns were the top three causes of severe burns in most studies (Figure 2). In four studies (from Finland, Spain, Turkey, and Slovakia), scalds were more prevalent than flames (up to 63%) [28,30,41,58]. In pediatric populations, scalds clearly dominate, accounting for 60% to 75% of all hospitalized burn patients, followed by flame and contact burns. Especially children younger than 2 years are at high risk for scalds, and the proportion of scalds is reported to be increasing over the years among pediatric populations [59,67,68,71]. In children presenting in the emergency department, scalds were most common (35% to 80%), followed by contact burns (13% to 47%), and flame burns (2% to 5%) [61-63,72]. In adult patients consulting the emergency department with burns, scalds were more prevalent than flame burns, although patients with flame burns are more frequently hospitalized [20,24,28].

Flame burns were more prevalent in men, whereas scalds and contact burns were more frequent in women [41,80]. Less frequent than flames, scalds, and contact burns are electrical burns, which were generally more frequent than chemical burns (Figure 2). In one Finnish pediatric study, electrical burns (20%) were more prevalent than contact burns (none) [60]. Two Turkish studies reported 17% to 40% electrical burns, which is supposed to be related to insufficient precautions and safety measures (as reported by the authors) [30,32]. Some specific causes of burns have been described separately in several studies (for example, sunburns (up to 5% of all burns, especially children)) [20,34,42,66,67,80], sauna (up to 26% of all burns in Finland) [58,70], and fireworks (up to 9% of all burns) [20,26,44,49,59,61,66,72,75].

The great majority of the burns are accidental, and especially in children, the majority occurred at home (80% to 90%) [2,14,41,42,44,59,66,71,75]. In the elderly, domestic burns (78% to 85%) [79,86,88] were followed by recreational accidents in 7% to 12% [80,83]. In adults, one third were work related [2,20,35,41]. The pediatric burns occurred mainly in the kitchen (75%), caused by hot food or beverages, with the bathroom as second most common location (mostly by immersion, leading to deeper and more extensive burns) [61,66-68]. Scalds in the elderly usually occurred in the bathroom (by immersion), in contrast to scalds in children, which usually occur in the kitchen [75,79,80,83,85,86].

Europe is considered to have the highest number of suicides in the world (World Health Organisation) [90]. However, only eight studies reported the number of self-inflicted burns: in three French (of which two are in the elderly), one Finnish, and one Spanish study, 3% to 6% of all burns were self-inflicted [14,44,58,80,83]. In three other studies (from the U.K., Turkey, and Slovakia), this percentage was less than 2% [27,40,41].

### Length of hospitalization

The mean length of hospitalization (LOS) in the general population with burn injuries was 7 to 33 days (median, 3 to 18 days) [1,2,19,25,29-32,50,52,56-58] and was reduced by 26% (1992 through 2007), as reported by one Norwegian study [56]. The average LOS in the pediatric population was 15 to 16 days (median, 10 to 12 days), and in the elderly, mean and median were reported as 18 to 26 days [61,65,67,73,80-84,88].

### Mortality and associated risk factors

In most hospitalized populations with severe burn injuries, the mortality rate lies between 1.4% and 18% (maximum, 34%). Several studies showed that older age, increasing TBSA age, and inhalation injury are the three major risk factors for mortality, although other variables have also been associated with a higher mortality risk [23,36,37,53].

**Figure 3:**
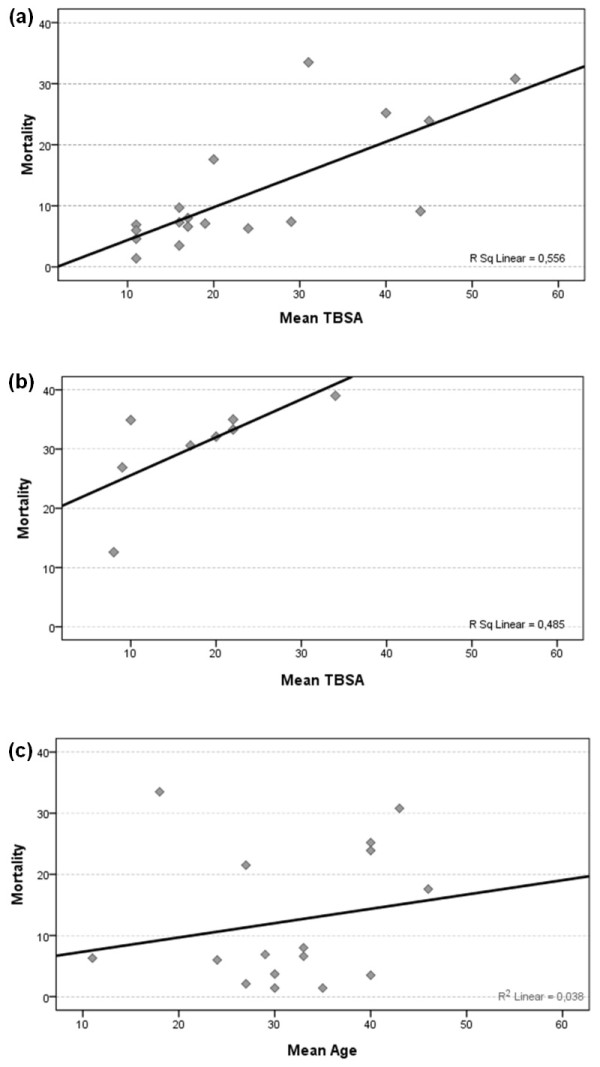
**The correlation between risk factors for mortality and mortality**. **(a) **Total and adult populations with severe burn injury: correlation between the mean total burned surface and mortality. (TBSA, total burned surface area). **(b) **Elderly populations with severe burn injury: correlation between the mean total burned surface and mortality. **(c) **Total and adult population with severe burn injury: correlation between the mean age and the associated mortality.

The mean TBSA in patients with severe burn injury was 11% to 24% and has decreased over the past decades, as reported in two studies [40,52]. The mean TBSA was higher among the deceased patients (44% to 50% overall; 73% in a pediatric study and 22% in an elderly population). In some studies, the average TBSA was remarkably higher (up to 55%), probably due to more strict admission criteria (for example, only intensive care patients, or only patients with a TBSA ≥30%), which was associated with higher mortality rates. The mortality increases considerably above a TBSA of 20% (Figure 3) [23,53]. The Pearson correlation test showed a positive correlation between the mean TBSA and mortality in the adult/overall age group (*r *= 0.741; *P *< 0.001), as well as in the studies discussing elderly populations (*r *= 0.696; *P *= 0.028; cf. correlation plot, Figure 3a, b), which clearly suggests a higher mortality when the TBSA (of the population) increases.

Another major risk factor for mortality is increasing age, which correlated noticeably with mortality, with 13% to 39% mortality among the cohorts of elderly patients. In contrast, a survival rate of 98% to 100% was reported in most pediatric series. When the adult and overall studies were analyzed together, a small positive correlation was found between age and TBSA (*r *= 0.195; *P *= 0.235) (Figure 3c). When the studies of the elderly population were also included in the analyses, a more-prominent correlation was found (*r *= 0.646; *P *< 0.001).

Besides age and TBSA, inhalation injury has repeatedly been associated with increased mortality (eight- to 10-fold higher [91]). Inhalation injury is due to smoke inhalation and is therefore especially prevalent in populations with a high proportion of flame burns [48,52]. The occurrence rate of inhalation injury is blurred by problematic diagnosis and hence lack of consensus definition. Some studies included all suspected inhalation injury; others, those confirmed by bronchoscopy or only those requiring mechanical ventilation [13,36,38,44,48]. Overall, inhalation injury occurred in 0.3% to 43% of all hospitalized patients with severe burn injury, and in 13% to 18% of the elderly with severe burn injury. Only two pediatric studies reported inhalation injury, in 3.3% and 69%, respectively [60,66]. No clear relation with mortality can be detected in these data.

Seven studies report a higher female mortality [17,28,31,32,36,42,53], but in seven other studies, no significant difference was found or even an increased male mortality [22,30,38,44,50,54,57]. In the elderly population, a significantly higher male mortality has been described [44,82]. Risk-adjusted mortality rates considering age and TBSA were, however, not provided, and therefore, no conclusions can be made about the relation between gender and mortality.

Flame burns have been associated with a higher mortality rate, but flame burns have also been associated with more-extended, deeper burns and the presence of inhalation injury [28,30,32,44,58].

Chronic diseases, including lifestyle risk factors such as chronic alcohol abuse and smoking, do compromise the prognosis of the patient with severe burn injury [36,47] and were present in 44% to 50% [79,81,84,86]. Co-morbidity was especially common among the elderly with severe burn injury (71% to 85%) [15,81,83,84,86]. Most frequent were cardiovascular (hypertension, ischemic heart diseases) and pulmonary diseases (chronic obstructive pulmonary disease), diabetes mellitus, and neurologic conditions [15,83,86]. Chronic alcoholism and psychiatric problems were present in 25% to 42% and 13% to 50%, respectively, of the deceased elderly with severe burn injury [61,65,67,73].

### Trends in mortality

The mortality decreased over the last 30 years (although the reporting of mortality is too heterogeneous to summarize) (Additional file 1). One Spanish study reported, for example, a reduction from 24% to 12% mortality, between 1992 and 1995 and 2001 to 2005 [53]; a Turkish study, from 38% to 30% (1988 through1992 versus 1993 through 1997) [32]; and a Dutch study reported a decrease from 7% to 5% between 1996 and 2006 [51]. A Danish study reported a decrease of mean mortality from four to three annual deaths [16]. The decrease in mortality was more apparent in the male population, as reported by one Swedish study [50], and was also more significant in patient groups of intermediate severity [52].

### Cause of death

Only a few articles report the cause of death, which was usually based not on autopsy results but on clinical presumptions. Early death (<48 hours) was mostly due to burn shock or inhalation injury [28,42,44,51,86]. Multiorgan failure was responsible for 25% to 65% of all burn deaths [28,32,42,51,81], and sepsis, for 2% to 14% [28,42,84,86]. Respiratory complications (pneumonia, ARDS, pulmonary embolism) are a major cause of death responsible for up to 34% among adults [16,28,42], and even up to 45% among the elderly [81,84,86]. Cardiac, renal, and cerebral complications each contribute to less than 5% of all deaths, but clear trends cannot be described because of the paucity of data. In one Turkish study, 45% of all deaths were ascribed to acute kidney injury [32].

### Socioeconomic status versus burn injury

Of all 76 studies, the great majority (89.5%) considered populations with a 'very high' HDI (68 studies). Only eight studies were published in countries with a 'high' HDI, and none, in countries with a medium HDI (Table 2) [12]. The 'very high' HDI countries are overrepresented, because 52% of the European countries have a 'very high' HDI; 37%, a 'high' HDI, and 4.3%, a medium HDI (Table 3).

**Table 3:** Distribution of studies by Human Development Index (HDI)

HDI	Number of studies	Number of countries^a^	Number of inhabitants (×10^6^)^a^
Very high	68 (89.5%)	24 (52.2%)	423 (52.2%)
High	8 (10.5%)	17 (37.0%)	377 (41.6%)
Medium	0	2 (4.3%)	50.1 (6.2%)
Low	0	0	0
Not known	0	3 (6.5%)	0.07 (0.0)

Total	76	46	810

HDI, Human Development Index. ^a^cf. Table 1.

Mainly because of the lack of studies from the less-developed European countries, and the often incomplete data, it is difficult to compare the impact of economy, standard of living, and so on, on the epidemiologic parameters discussed earlier. Most remarkable are the high prevalence of electrical burns in the three Turkish studies (13% to 40%), especially because only one of the other studies reports a prevalence of electrical burns higher than 8% (a pediatric study from Finland [60]). The male predominance was also less apparent (or even absent) in the 'high' HDI countries, because three of four studies reporting the lowest proportions of men come from 'high' HDI countries (33% to 54%) (studies considering the elderly population were not taken into account) [27,30,54]. Insufficient data are available to assess the influence of HDI on other epidemiologic parameters, which is also because of the multifactorial relations between severity, incidence, outcome, and so on.

## Discussion

This study provides an overview of the epidemiology of severe burn injury in Europe, based on observational studies published in the last 25 years. Despite the lack of a large-scale European registration of burn injury, some strong conclusions can be made. These include a decrease in incidence and mortality, a male predominance, and age-related etiology patterns. The decreasing incidence is almost certainly related to increased awareness of hazardous situations through prevention campaigns and better regulations for electronic equipment. Increased insight into the pathophysiology of burn injury has undoubtedly contributed to improvements in therapy, such as fluid resuscitation, infection prevention, and wound care, leading to a higher survival rate. A decrease in severity of the burns should also be kept in mind, as a decrease in TBSA was noted in two studies. Considering the etiology, flame burns are the most frequent cause among adults, and scalds, among children, but cultural and socioeconomic differences do have a major influence. Although a decreasing incidence of burn injury has been described, the great majority of the burns remain accidental, and therefore are preventable, especially in children. Probably at least as important as further improvements in burn management, prevention of burn injury is crucial to decrease the morbidity, mortality and economic burden caused by severe burn injury [2].

Although this study is based on a cohort of almost 200,000 patients hospitalized with burn injury (which is, as far as we know, the largest ever described), this study has several limitations. Most included studies were small, multicenter studies of retrospectively collected data, but especially the heterogeneity of study populations hampers comparisons. Some differences between studies are probably due to socioeconomic, logistic, or even cultural differences (for example, in cooking and saunas). For instance, the number of burns due to electricity is alarmingly high in Turkey, which is reported to be caused by insufficient information about the dangers of electricity; or even more likely by unsafe electrical appliances and electricity distribution. The variation in the severity of the population with severe burn injury (for example, TBSA) could be explained by differences in the accessibility of the European burn units (differences in the transport network, and geographic distribution and number of the burn units), the admission criteria of the burn units, and/or differences in age distribution or other demographic characteristics.

The differences between the populations with burn injury will also be related to differences in the standard of living and economy. Unfortunately, the quantity and quality of research is often related to the economy and standard of health care, because research is possible only if resources and qualified personnel are available. When compared with studies from other highly industrialized countries in North America, Australia, and Asia, this study provided similar results, whereof the decreasing mortality and incidence, risk factors for mortality, and distribution of etiology are among the most frequent reported parameters [92-95].

It would be interesting to compare the epidemiology of burn injury between highly industrialized countries and developing countries, but national registration is not even established in several highly developed (European) countries, and probably completely absent in several developing countries. For this study, we attempted to analyze the differences between the most-developed European countries and the 'less' developed countries (although the differences considering the human development statistic appeared to be rather small). Because the most developed countries were overrepresented, and thus insufficient data were available, it was not possible to draw strong conclusions considering the standard of living and burn epidemiology. Most remarkable was the absence of a male predominance and higher proportion of electrical burn injury in the least developed European countries. It can be expected that the differences (in standard of living, health care, and so on) between all European countries will diminish even further.

Another limitation of this study is the absence of uniformity resulting in often suboptimal reporting and analyses of data, with other classifications and definitions for etiology, inhalation injury, and so on. For example, the cut-off values for our three age categories (children, adults, and elderly) posed no problem for the pediatric population (younger than 15 to 16 years) but ranged from 60 to 75 years for the elderly.

This study cannot provide a clear answer to the often-questioned gender-related differences in outcome, because no risk adjustment is performed in the individual studies to exclude the influence of effect-modifying factors such as TBSA, age, and etiology. The geographic distribution of the available studies also makes extrapolation to the whole of Europe questionable. We aimed at a description of all European countries, but some regions were overrepresented (half of the studies were published in only four different countries), and from certain regions, no data were available at all. This might be due to the language restrictions of our search (we included studies in only English, French, and Dutch), but also due to the predominance of the English language as the international scientific language. The included languages are native languages in only a minority of the European countries (especially located in Western Europe), which may hamper publication of studies from non-native English-speaking countries. However, the impact of our language barrier will probably be limited, because the inclusion of French and Dutch contributed to only three additional articles, and 82% of all studies considered populations in which English was not their native language.

Hence, the further implementation of national and preferably also international registration systems with consensus definitions of hospitalized patients with severe burn injury will facilitate research through more extensive databases and hence will enable detection of possible relations between risk factors. Consequently, a more accurate registration and description of the population with severe burn injury may allow improved targeting of prevention campaigns and cost-effectiveness of total burn care. Therefore, we promote the development of a European-scaled registration network that will provide detailed epidemiologic insights and will allow bench-marking and quality of burn care.

## Conclusions

Although this study is based on a very heterogeneous group of populations from all over Europe, it is based on a very large cohort of patients covering a period of 25 years. Several strong conclusions can be made about age-related etiology patterns and gender distribution, and (trends in) incidence and mortality. National and international registration of burn injuries will enable further epidemiologic research, and will certainly lead to better targeted prevention campaigns and a better, cost-economic multidisciplinary burn treatment.

## Key messages

• Severe burn injuries (requiring hospitalization) still occur often and have a high impact on morbidity and mortality. In some countries, a decreasing incidence is noted over time.

• Half of the patients are younger than 16 years, and up to 75% of the victims are male patients (except in the elderly population).

• Flame burns and scalds are the most frequent causes of burns among all age groups.

• Mortality varies considerably among different populations (range, 1.4% to 34%, with a decreasing trend over time), and clearly correlates with an increasing mean total burned surface area.

• National and international registration of epidemiologic data of populations with burn injuries should be promoted. Consensus definitions (for example, inhalation injury) are, however, obligatory to compare different populations and will subsequently improve burn care.

## Abbreviations

LOS: Length of stay (hospitalization); TBSA: total burned surface area.

## Competing interests

The authors declare that they have no competing interests.

## Authors' contributions

All authors made substantial contributions to the conception and design. NB, EH, and SB selected the literature and performed the statistical analyses. The manuscript was drafted by NB, helped by SB and EH, and the manuscript was critically revised by SM and DV. All authors have read and approved the final manuscript.

## Supplementary Material

Additional file 1 **Overview table of all 76 included studies**. BOBI, Belgian Outcome in Burn Injury Study Group; P, Prospective; R, Retrospective; ED, Emergency Department; n.r., not reported; S, Survivors; NS, Non-survivors; M, male; F, Female; *All national (paediatric) burn units. **Nationwide data: based on national registers or registration systems and so on (may also include hospitals without specialized burn unit); °Pediatric surgical departments; #also includes patients with secondary diagnosis of burns; +only patients with burns and inhalation injury. Incidence trends reported as increase (↗) or decrease (↘) in incidence (and/or annual number of admitted patients). Mortality trends reported as increase (↗) or decrease (↘). Click here for file
